# Neem ( *Azadirachta indica* ): A Miracle Herb; Panacea for All Ailments

**DOI:** 10.1002/fsn3.70820

**Published:** 2025-09-01

**Authors:** Tabussam Tufail, Huma Bader Ul Ain, Aiman Ijaz, Muhammad Adnan Nasir, Ali Ikram, Sana Noreen, Muhammad Tayyab Arshad, Muhammed Adem Abdullahi

**Affiliations:** ^1^ College of Pharmaceutical Science, Zhejiang University of Technology Hangzhou China; ^2^ University Institute of Diet and Nutritional Sciences, the University of Lahore Lahore Pakistan; ^3^ School of Food Science and Engineering Yangzhou University Yangzhou China; ^4^ Department of Allied Health Sciences The University of Chenab, Adjacent to Chenab Bridge Gujrat Pakistan; ^5^ University Institute of Food Science and Technology, the University of Lahore Lahore Pakistan; ^6^ Functional Food and Nutrition Program, Faculty of Agro‐Industry Prince of Songkla University Songkhla Thailand; ^7^ Department of Food Science and Postharvest Technology Jimma University College of Agriculture and Veterinary Medicine, Jimma University Jimma Ethiopia

**Keywords:** antioxidant, *Azadirachta indica*, herb, neem

## Abstract

The neem tree ( *Azadirachta indica* ), native to the Indian subcontinent, has recently gained global recognition because of its extensive therapeutic qualities. It contains a high concentration of antioxidants and other valuable active substances including azadirachtin, salannin, nimbidin, nimbolinin, nimbidol, nimbin, and quercetin, which are extracted from various plant parts. It has been widely utilized in Ayurveda, Unani, and Homeopathic treatments and has gained significant attention in modern medicine. Traditionally, neem leaves, flowers, seeds, fruits, roots, twigs, and bark have been used to treat fever, infection, skin conditions, and dental problems. The immunomodulatory, antiviral, anti‐ulcer, antioxidant, anti‐inflammatory, antihyperglycemic, antifungal, and anti‐carcinogenic properties of neem and its components are well known. This comprehensive review highlights the diverse phytochemicals derived from neem leaves, extraction techniques, and their medicinal value in treating of multiple medical conditions. This miraculous plant has significant potential for enhancing its effectiveness. People have appropriately referred to it as a “natural remedy for numerous illnesses.”

## Graphical abstract

This comprehensive review highlights the diverse phytochemicals derived from neem leaves, extraction techniques, and their medicinal value in the treatment of multiple medical conditions. This miraculous plant has significant potential for enhanced effectiveness. People have appropriately referred to it as a “natural remedy for numerous illnesses.”

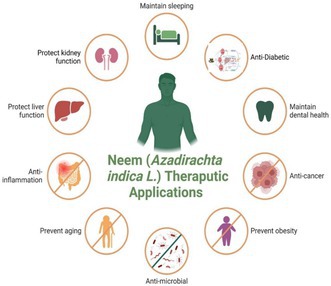

## 1 Introduction

The *Azadirachta indica* plant is sometimes called the Neem or Margosa tree. Ayurvedic, Homeopathic, and Unani treatments have been widely used. For over 2000 years, the Indian subcontinent has recognized *Azadirachta indica* , popularly known as neem, as one of the most adaptable healing plants with a broad range of biological activities (Reddy and Neelima 2022). Uzzaman (2020) stated that a therapeutic plant has pharmacological action to treat ailments instead of an edible plant utilized in everyday life as a meal. *A. indica* has been classified into two species: Indica Azadirachta (A. Juss.), local to the subcontinent of India, and Azadirachta excelsa (Jack), exclusive to Indonesia and the Philippines. It is native to the nations of the Indian subcontinent, which include Bangladesh, Sri Lanka, Nepal, India, Pakistan, and the Maldives. It is a member of one of two species of the genus *Azadirachta* (Upadhayay and Vigyan 2014).

A state of “excellent health” is described in Sanskrit as “Nimba” which eventually evolved into “Neem.” The tree is referred to as “Sarvaroga nivarini,” which means “the remedy for all ailments” or “the panacea for all ills.” Neem is referred to as “Arishtha” in Ayurveda, which means “reliever of illnesses.” Due to the tree's therapeutic capabilities, it is still referred to as a “village pharmacy,” “Divine tree” and “nature's drugstore” in India (Devi and Sharma 2023). *A. indica* , the Persian designation for neem, translates to “the free tree of India,” highlighting its resistance to parasites and diseases. The UN named the neem tree the “Tree of the 21st Century” for its extraordinary features. The UN named neem “Tree of the 21st Century.” US National Academy of Sciences released “Neem: A Tree for Addressing Global Challenges” in 1992 (Moin et al. 2022).

In Ayurveda, Unani, Homeopathy, and modern medicine, neem components are used to treat viral, metabolic, or neoplastic illnesses. The leaves, seeds, blossoms, and bark of this tree are widely used for various applications. Numerous phytochemicals have been extracted from different plant parts, including triterpenes, gallic acid, nimbins, saponins, catechins, limonoids, flavonoids, phenols, and glycoproteins (Sandhir et al. 2021). The leaves also contain a variety of active ingredients, but the most important active constituent is azadirachtin, while the others are sodium nimbinate, gedunin, salannin, quercetin, nimbin, nimbidin, and nimbidol (Joshi and Prabhakar 2021).

Previous studies have shown that the crude extract of neem leaves has strong hypoglycemic, hypolipemic, hepatoprotective, and hypertensive properties. Neem has anti‐inflammatory, antioxidant, antiviral, and antidiabetic effects. Numerous effects have been investigated, including anticancer, antibacterial, antiparasitic, antipyretic, immunomodulatory, antimicrobial, antifungal, hepatoprotective, and gastroprotective (Asghar et al. 2022). Particular focus should be placed on employing non‐toxic herbal items to control illnesses in humans and animals. Additionally, care should be taken to assess the safety of various neem and neem compounds. There is a ton of potential for this miraculous plant to be used effectively. The significance of bioactive compounds in the neem and the inhibition and treatment of illnesses are briefly discussed in this article.

## 2 Botanical Description

**FIGURE 1:**
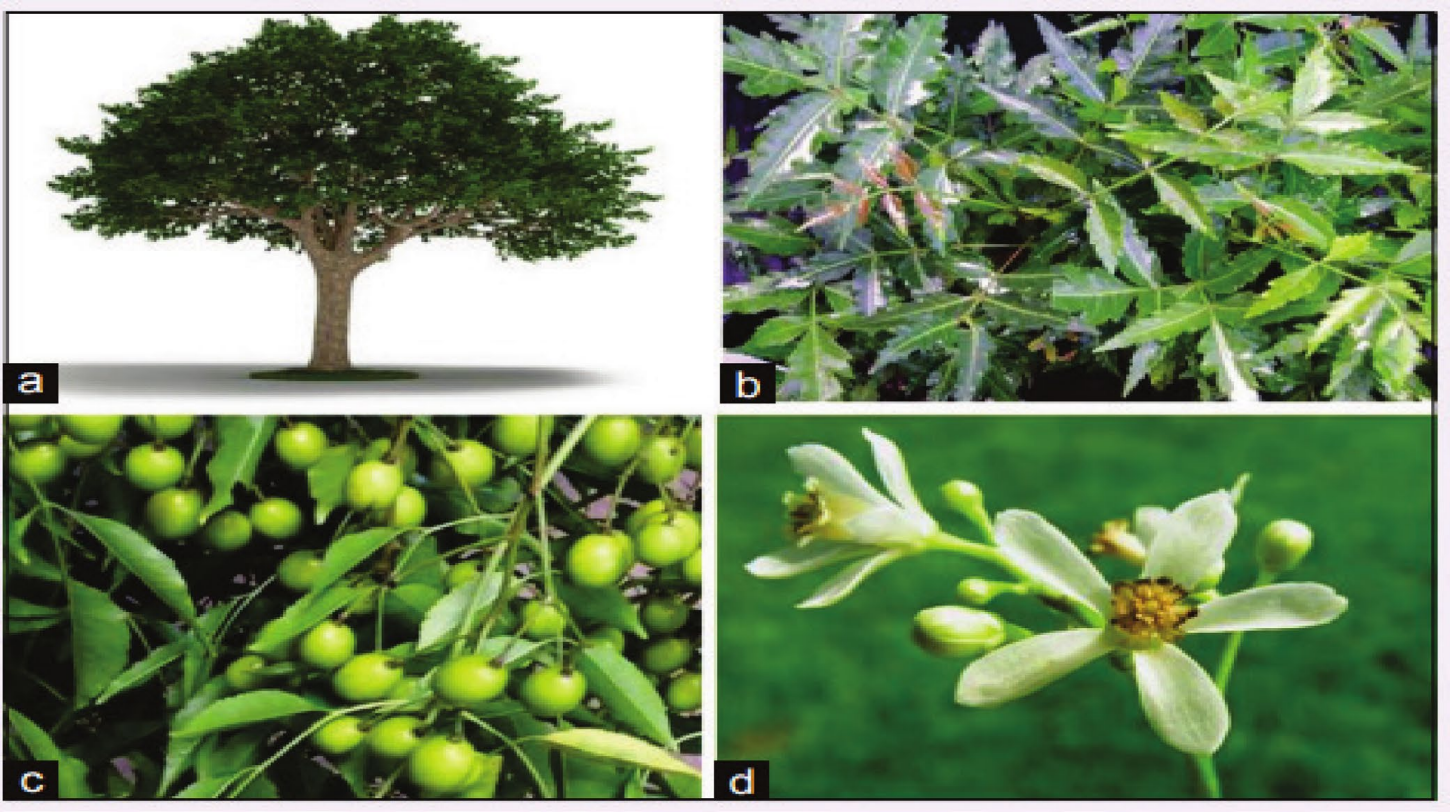
Neem tree and its different parts (A–D).

It is grown most frequently in tropical and semi‐tropical climates. This tree has a rough gray bark. Neem trees thrive in regions with low rainfall. The tree height is approximately 12–15 m and occasionally 25–35 m. A flowering plant typically begins bearing fruit after 3–5 years. Within 10 years, trees begin to bear fruit (Islas et al. 2020). Neem trees can survive 150–200 years and increase the size of shade trees with thick, rounded canopies. The taste of the entire tree is harsh—green, with asymmetrical leaves and blunt serrations (Figure 1). The leaves reached a length of 30 cm. Each leaf had 10–12 serrated leaflets. They are 2.5 cm broad and 7 cm long (Bhamare et al. 2020).

The blossoms are white and have a fragrant aroma, particularly at night. Flowers bloom twice a year, in April to May and August to September. A neem tree produces 20–40 kg of fruit each year. Its fruit, called nimboli, is tiny, oblong, and smooth. The unripe fruit is dark and bitter, but the ripe fruit is yellow and slightly sweet. Taxonomical classification and vernaculars of neem (Table 1) (Semere 2023). Table 1 depicts the taxonomical classification and vernaculars of neem.

**TABLE 1:** Taxonomical classification and vernaculars of neem (Uchegbu et al. 2011; Quraishi et al. 2018).

Vernaculars of neem		Taxonomic positions of neem	
English	Indian Lilac, Margosa Tree	Kingdom	Plantae
Urdu	Neem	Order	Rutales
India	Indian Lilac Tree	Suborder	Rutinae
Hindi	Balnimb, Nim, Nimb	Family	Meliaceae
Indo China	Sau dau, Sdao, Xoan dau	Subfamily	Melioideae
Arabic	Al Shurisha	Tribe	Melieae
		Genus	Azadirachta
		Specie	Indica
		Latin	*Azadirachta indica*

## 3 Utilization of Particular Neem Parts

### 3.1 Flowers

Most parts of the neem tree are unpleasant, except for its flowers. Neem flowers are delicate, white with grayish buds, refreshed, and too beautiful to consider eating. The flowers bloom twice, once in the evening and once late at night, and have a lovely, almost mystical, jasmine‐like smell in the evening. They are dispersed directly under the trees during rainfall. These neem flowers, known as Vepampoo in Tamil, can be used fresh, dried, or powdered (Prakash et al. 2021). They are frequently used in the South to prepare various foods, including lentils, bloom rice, pachadi, and rasam, and that is only the beginning. They are typically dry‐boiled and used as garnishes in cuisine. Neem flowers are a natural remedy for intestinal worms, vomiting, nausea, and anorexia (Table 2) (Sharma and Paliwal 2021).

**TABLE 2:** *Azadirachta indica* plant parts and their utilization.

Parts	Form	Utilization	References
Leaf	Traditional medicine	Neem leaves have been employed in traditional medicine for ages due to their medicinal qualities They may have antiviral, antifungal, antibacterial, and anti‐inflammatory properties. Neem leaf extract treats skin conditions such as eczema, psoriasis, and acne Additionally, people take it internally to treat conditions like high blood pressure, diabetes, and digestive issues	Reddy and Neelima (2022)
	Herbal preparation	Neem leaves can undergo dehydration and pulverization processes, making them useful for making capsules or tea People frequently consume neem leaf tea for its astringent flavor and potential therapeutic benefits	Kalaskar et al. (2021)
	Agricultural use	Neem leaves include inherent properties that make them effective as a pesticide and repellant against insects They can be utilized in diverse forms, such as a spray made from extracting the essence of leaves, to manage pests in agriculture.	Gupta (2022)
Seed	Oil production	Neem seeds are the source of Neem oil, a versatile material used in cosmetics, soaps, shampoos, and medications Neem oil is rich in fatty acids and contains chemicals such as azadirachtin, which has insecticidal qualities	Chaudhary et al. (2021)
	Insecticidal use	Neem oil is a biologically derived substance that effectively controls insects and fungi. This solution exhibits efficacy against various pests, encompassing aphids, caterpillars, mites, and leaf miners It hinders the growth and maturation of insects, making it a widely favored option for organic farming	Muhammad and Kashere (2020)
Bark	Medicinal purposes	The Neem tree bark includes a variety of bioactive chemicals, including quercetin and nimbidin, which possess antipyretic, antimalarial, and analgesic activities Historically, it has been employed in Ayurvedic medicine to address conditions such as fever, malaria, and discomfort	Ogidi et al. (2021)
	Oral care	Herbal toothpaste and mouthwash contain neem bark The antibacterial characteristics of this substance aid in preserving oral hygiene and averting gum disorders such as gingivitis	Kalaskar et al. (2021)
	Industrial use	Neem bark contains tannins, which are utilized for tanning leather and coloring fabrics. Tannins can form bonds with proteins, which is beneficial in the tanning process because it facilitates the transformation of animal hides into leather	Das et al. (2020)
Twig	Oral hygiene	Traditional oral hygiene procedures use Neem twigs as natural toothbrushes Chewing on neem twigs or using them as toothbrushes is believed to help prevent tooth decay, gum disease, and bad breath Neem twigs possess antibacterial characteristics that can impede the proliferation of microorganisms in the oral cavity	Kishore et al. (2023)
Flower	Medicinal use	Traditional medicine commonly uses neem flowers due to their therapeutic characteristics. They offer digestive benefits and serve as a blood purifier Neem flower infusions or extracts are occasionally employed to treat gastrointestinal problems such as constipation, bloating, and indigestion	Islas et al. (2020)
	Anthelmintic properties	Neem flowers possess anthelmintic qualities, which aid in expelling intestinal worms from the body. Traditional treatments for deworming may use infusions or decoctions made from neem blossoms	Rahaman et al. (2022)

According to a study by Gbotolorun et al. (2008), in 80% of rats, diestrus was prolonged, altering the estrous cycle. Neem flower resulted in a substantial (*p* < 0.05) decrease in ova lost during estrus in rats administered the extract at 9 a.m. on proestrus. Neem flowers had no anti‐implantation/abortifacient or teratogenic effects on rats. The estrous cycle in Sprague–Dawley rats was interrupted by neem flower alcohol. It partially blocks ovulation and might become a female contraceptive. Table 2 depicts the *Azadirachta indica* plant parts and their utilization.

### 3.2 Leaves

Neem leaves have excellent healing properties. In addition to being useful for controlling diseases and pests, they can benefit animals when mixed with other grains. In some parts of India, especially in the southern Indian states, neem leaves are used as fertilizer in rice fields (Subapriya and Nagini 2005).

Neem leaves are sometimes used as a growth medium in tomato and tobacco farms. By applying them to plant roots to retain moisture, they can be utilized to effectively eradicate weeds in all environments. Neem leaves may also be used to keep pests out of wool and silk clothing that is being preserved (Latif et al. 2020). Thai people take the young leaves and blooms of Siamese neem as bitter tonic vegetables. The plant extract was found to be non‐toxic. In their study, Sithisarn et al. (2007) showed that extracts from Siamese neem tree leaves effectively prevented the production of hypervalent iron or efficiently deactivated the hypervalent state.

In another study conducted by Onyimonyi et al. (2009), broiler performance and economic variables were analyzed. Sun‐dried Neem Leaf Meal was fed to broilers in various amounts. The study contained 90 Ross 2‐week‐old unsexed broilers. The birds were randomly assigned to five 18‐bird treatment groups. For treatments 1, 2, 3, 4, and 5, NLM was added at 0%, 0.5%, 1.0%, 1.5%, and 2%. All three treatments were done twice throughout the experiment. Each replication had nine birds. The experimental design was completely random. The results showed that treatment significantly affected AFBW, ADG, ADFI, and FCR.

### 3.3 Neem Cake

Neem cakes are dynamic and have several applications. They can be used as a normal insecticide, compost, and animal feed. Combined with urea before being applied to the field, they provide natural nitrogen while impeding nitrification. Using a 90:10 ratio of neem‐coated urea can prevent up to 30% of the total synthetic nitrogen requirement of crops from wasting. This results in lower production costs of horticulture. In India, neem cake is typically used as a fertilizer for sugarcane, vegetables, and other commercial crops (de Sá Leitão et al. 2022).

According to Benelli et al. (2015), neem cake, a low‐cost byproduct obtained from neem oil extraction, is a crucial source of toxic metabolites in mosquitoes. This review focuses on four main aspects: (i) recent advancements in neem cake metabolomics, specifically regarding nor‐terpenoids and related compounds; (ii) the toxic effects of neem cake on the eggs, larvae, and adult mosquitoes of Aedes, Anopheles, and Culex species; (iii) the unintended effects of neem cake on non‐target vertebrates; and (iv) the ability of neem cake to deter mosquito females from laying eggs. Neem cake might be an environmentally friendly and cost‐effective chemical source for developing more advanced and secure control methods against mosquito vectors. Neem cake is a cost‐effective and readily accessible natural resource that shows promise for developing a new bioinsecticide, particularly in developing countries (Nicoletti et al. 2010). Another study conducted by Chandramohan et al. (2016) revealed that the methanolic fractions of neem cake can serve as a novel and cost‐effective source of potent chemicals to combat An. culicifacies, malarial vectors that are prevalent in rural areas.

### 3.4 Twigs and Bark

Neem belongs to the Mahogany family and it grows rapidly. The tree has an abundance of branches. Rural individuals and peasants use the diverse health properties of neem twigs for dental hygiene. Researchers have extracted and separated several compounds from various plant parts. Neem twigs contain fibrous materials (Raja and Devarajan 2023). As an Asian native, you probably witnessed someone biting into a neem branch. Neem twigs have been used as temporary toothbrushes for a long time. They eliminate bacteria, maintain the soluble components in the saliva, control microbes, cure inflamed gums, and make teeth whiter. The twig also separates into threads resembling fibers that degrade and prevent plaque (Reddy and Neelima 2022).

Neem twigs can be used as oral deodorants, toothache analgesics, and for dental hygiene. The neem bark is an antibacterial and deodorizing agent. Among the various phytochemical components of the neem plant are nimbidin, nimbin, nimbolide, gallic acid, epicatechin, catechin, and margolone. All these compounds showed a strong antibacterial effect (Lakshmi et al. 2015). The study conducted by Adyanthaya et al. (2014) showed antibacterial efficacy in neem twig extract against cariogenic and periodontal disorders. For oral healthcare, bioactive components in the extract should be separated and identified.

### 3.5 Neem Oil

Neem oil comes from the Meliaceae tree *Azadirachta indica* Juss. This Indian plant is now respected worldwide for its contribution to phytochemical production for human health and pest control (Forim et al. 2014). Rapidly growing, tiny to medium‐sized evergreen *Azadirachta indica* with sprawling branches. It thrives in elevated temperatures and suboptimal soil conditions. Adult leaves exhibit a vibrant green hue, while young leaves display shades of red to purple. The petiole, lamina, and base of the leaf are connected to the stem. Two basal stipules are leaf‐like lateral formations (Aneesa 2016).

Neem oil is composed of several bioactive components. The primary components are triterpenes, specifically limonoids. The most significant pest is azadirachtin, which is responsible for approximately 90% of the impact on most pests (Roy and Saraf 2006). Additional constituents include meliantriol, nimbin, nimbidin, nimbinin, nimbolides, fatty acids (oleic, stearic, and palmitic acids), and salannin. The primary neem product is the oil obtained from seeds using various extraction methods. Although other components of the neem tree have lower levels of azadirachtin, they are utilized for extracting oil and as a food source for insects (Saxena et al. 2018).

The medicinal characteristics of neem oil, extracted from neem seeds, make it a fantastic ingredient in cosmetics and other high‐quality products such as hand soap, cleansers, and hair oil. It is recognized as an excellent mosquito repellent that can cure a wide range of skin diseases (Chaudhary et al. 2021). Neem can also be used by the body when coupled with coconut oil. It is believed that neem oil is promoted to children in India as a type of cure. Neem oil is a fantastic Ayurvedic healer that protects various plants. It can also be found in creams, lotions, cleansers, and other soothing products (Amra et al. 2022).

## 4 Bioactive Profile

**FIGURE 2:**
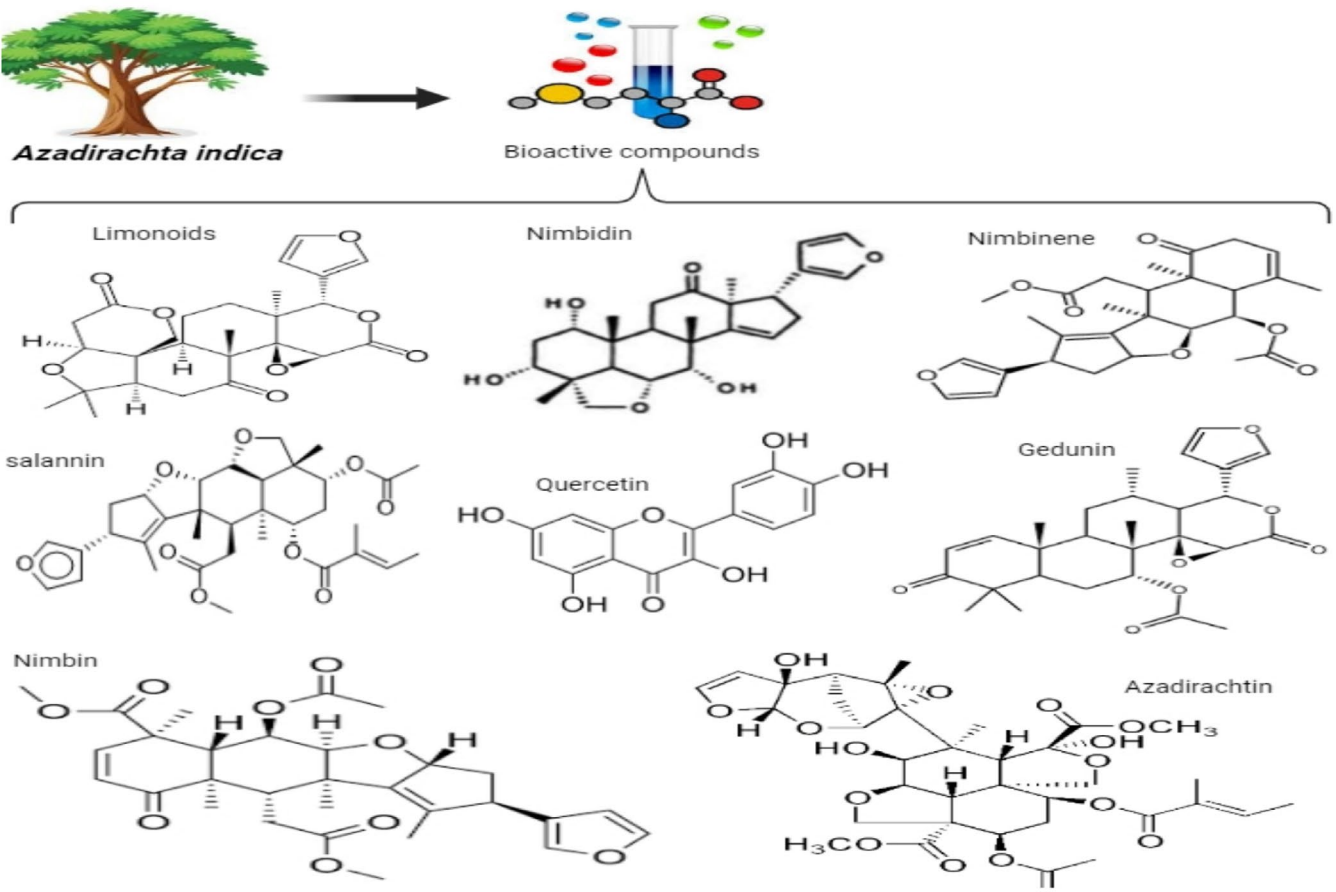
Chemical structure of main bioactive compounds of neem.

*Azadirachta indica* L. is a rich source of many compounds (Figure 2). Azadirachtin is the most important active constituent. Sodium nimbinate, nimbidol, nimbin, nimbolinin, nimbidin, gedunin, salannin, quercetin, ascorbic acid, n‐hexacosanol, amino acids, 6‐desacetylnimbinene, nimbandiol, nimbolide, 7‐desacetyl‐7‐benzoylazadiradione, 7‐desacetyl‐7‐benzoylgedunin, 17‐hydroxyazadiradione, and nimbiol are among the substances present in leaves (Sarkar et al. 2021). Polyphenolic flavonoids, that is, ß‐sitosterol and quercetin, have been recognized to have antifungal and antibacterial properties and were isolated from fresh neem leaves (Cheng et al. 2024). Gedunin and azadirachtin are two important components of neem seeds (Table 3) (Joshi and Prabhakar 2021).

**TABLE 3:** Neem medicinal ingredients and their phytochemicals and benefits.

Medicinal ingredient	Phytochemical	Effects	Uses and benefits	References
Azadirachtin	Limonoids	Antifeedant	Azadirachtin functions as an insect‐feeding deterrent, rendering it a valuable natural insecticide	Qin et al. (2020)
		Insecticidal	It hinders the growth and development of insect larvae and has demonstrated effectiveness against a broad spectrum of pests	Kilani‐Morakchi et al. (2021)
		Anticancer	Studies indicate that azadirachtin demonstrates cytotoxic properties against cancer cells, suggesting its potential as an anticancer drug	John and Raza (2023)
Nimbin	Tetranortriterpenoids	Anti‐inflammatory	Nimbin exhibits anti‐inflammatory properties, rendering it valuable for addressing diverse inflammatory ailments like arthritis and skin problems	Sudhakaran et al. (2022a, 2022b)
		Antimicrobial	It has a wide range of antibacterial action against bacteria, fungi, and viruses, which explains its traditional application in wound healing and infection management	Sudhakaran et al. (2022a, 2022b)
		Antipyretic	Historically, people have used nimbin for its antipyretic properties, which help to lower fever	Oktavia and Ifora (2022)
Nimbidin	Tetranortriterpenoids	Antihyperglycemic	Nimbidin has exhibited hypoglycemic properties, which can be advantageous in treating diabetes by reducing blood glucose levels	Patil et al. (2022)
		Antioxidant	It demonstrates potent antioxidant properties, neutralizing free radicals and safeguarding cells against oxidative harm	HNSKTavva et al. (2023)
		Wound healing	Nimbidin facilitates wound healing and skin regeneration, rendering it beneficial in managing lacerations, thermal injuries, and other dermal traumas	Thomas et al. (2024)
Quercetin	Flavonoids	Anti‐inflammatory	Quercetin shows strong anti‐inflammatory properties by suppressing the production of inflammatory mediators and blocking the pathways involved in inflammation. This ability may be beneficial in reducing symptoms of inflammatory disorders like allergies and arthritis	Azeem et al. (2023)
		Antiviral	Evidence has demonstrated that it can impede the reproduction of certain viruses, such as respiratory viruses like influenza, potentially diminishing the intensity and length of viral illnesses	Di Petrillo et al. (2022)
		Antioxidant	Quercetin is a potent antioxidant that eliminates harmful free radicals, safeguarding cells from oxidative harm and diminishing the likelihood of chronic ailments like heart disease and cancer	Ersoz et al. (2020)
β‐Sitosterol	Phytosterols	Cholesterol‐lowering	β‐Sitosterol reduces LDL cholesterol levels by blocking cholesterol absorption in the intestines, potentially decreasing the likelihood of developing cardiovascular disease	Athraa (2022)
		Anti‐inflammatory	It has anti‐inflammatory effects that can relieve symptoms of inflammatory disorders such as arthritis and improve joint health	Jain et al. (2020)
		Prostate health	Research has explored the potential of Beta‐sitosterol in promoting prostate health and alleviating symptoms of benign prostatic hyperplasia (BPH)	Iman et al. (2022)
Kaempferol	Flavonoids	Antioxidant	Kaempferol is a powerful antioxidant that eliminates harmful free radicals and decreases oxidative stress, possibly protecting against chronic illnesses like cancer, diabetes, and neurological disorders	Reddy and Neelima (2022)
		Anti‐inflammatory	It demonstrates anti‐inflammatory properties by blocking the action of enzymes and cytokines that cause inflammation. This makes it useful for treating inflammation‐related illnesses like arthritis and inflammatory bowel disease	Biswal et al. (2021)
		Cardiovascular activity	Cardiovascular benefits of kaempferol include lowering blood pressure, improving blood vessel function, and reducing the risk of heart disease and stroke	Nwanekezie et al. (2023)
Azadirachtol	Tetranortriterpenoids	Antimicrobial	Azadirachtol demonstrates a wide range of antibacterial action against bacteria, fungi, and protozoa, which makes it valuable for treating several infectious disorders	Falana and Nurudeen (2020)
		Anti‐inflammatory	It has anti‐inflammatory characteristics that can decrease inflammation and alleviate symptoms of arthritis and skin ailments	Awadh et al. (2022)
		Wound healing	Azadirachtol enhances wound healing by expediting the generation of fresh skin tissue and diminishing the likelihood of infection, rendering it highly beneficial in wound care	Abbas et al. (2020)
Gedunin	Tetranortriterpenoids	Antimalarial	Gedunin has demonstrated potent antimalarial efficacy by effectively suppressing the growth and replication of Plasmodium parasites, the primary causative agents responsible for malaria	Braga et al. (2020)
		Anti‐inflammatory	It demonstrates anti‐inflammatory characteristics that can aid in diminishing inflammation and relieving symptoms of inflammatory ailments like arthritis and asthma	Sarkar et al. (2021)
		Antitumor	The potential anticancer properties of Gedunin have been investigated, including its ability to suppress the development and proliferation of cancer cells and induce apoptosis. These findings imply that Gedunin can potentially be a therapeutic agent for cancer treatment	Nagini et al. (2024)
Salannin	Limonoids	Insecticidal	Salannin functions as a natural pesticide, deterring and impeding the development of several insect pests, rendering it highly important in agriculture and pest management	Juma et al. (2022)
		Antifungal	It demonstrates fungicidal capabilities against a broad spectrum of fungi, aiding in the management of fungal diseases in both plants and humans	Joshi and Prabhakar (2021)
		Antimicrobial	Salannin exhibits bactericidal properties against bacteria and other pathogens, which explains its traditional application in promoting wound healing and controlling infections	Shishupala (2024)

Nimbin (a triterpenoid isolated from neem) possesses fungicidal, antipyretic, antihistamine, and antiseptic effects. Furthermore, nimbin possesses properties that help reduce inflammation and oxidative stress by limiting the production of oxygen species (ROS), thus minimizing damage, as discussed by Kumari and Singh (2024). An in‐depth study of the composition of the oil extracts revealed high concentrations of flavonoids, triterpenes, and saponins, which are commonly found in this popular herb (Luo et al. 2024). In contrast, according to Nagano and Batalini (2021), nimbin and catechins are present in large amounts. Other compounds identified in neem extracts include acids, limonoids, terpenoids, tannins, reducing sugars, alkaloids, and catechins, as highlighted by Bolaji et al. (2024). In animals, neem leaf glycoprotein (NLGP) modulates the immune system. It may come from tree leaves. According to Singh et al. (2022), NLGP may have the potential to inhibit tumor growth.

Recent findings by Wijanarko and Rifa'i (2020) on leaf extract analysis encompassed the methanolic extracts. The results revealed the levels of saponins, glycosides, and tannins, specifically in the aqueous extracts. The highest quantities of alkaloids, tannins, and flavonoids were detected in methanolic extracts. This demonstrates the wide range of readily available compounds but intriguingly places a lot of emphasis on the extraction process. Proline, a current therapy for neurological disorders, including Alzheimer's disease, Diabetes Mellitus Type 2, Parkinson's disease, and polycythemia, was found at significant concentrations in the biochemical examination of neem leaf extracts (Rahmah et al. 2024). Table 3 depicts the neem medicinal ingredients and their phytochemicals and benefits.

## 5 Extraction Methods

Neem oil was extracted using an ancient technique that involves placing crushed seed kernels in a water container and fetching the oil that floats to the surface. The quality was affected by the low yields. Neem oil was extracted commercially using a steam distillation method with petroleum ether (boiling point of 60°C–80°C). Ordinary distillation was used to separate the oil from the filtrate at 70°C. The oil was then placed in a round‐bottom flask and heated in a water bath for 20 h at 60°C–70°C a rotating vacuum evaporator to remove any remaining solvent. n‐Hexane and ethanol can also be used as solvents for oil extraction. Temperature, oxidation, hydrolysis, lipase enzymes, and unfavorable chemicals in the oil impact its quality (Kumar et al. 2016).

Organic solvents, such as ethers and dimethyl sulfoxide, were utilized to obtain highly concentrated extracts. Utilizing supercritical carbon dioxide flow at high pressures and temperatures may increase the concentration. To be used as a nutraceutical, neem must first remove its bitter active ingredients because they are abundant in the plant. Using water, methyl ethyl ketone, ethanol, methanol, methyl tert‐butyl ether, or their azeotropic mixtures as solvents allows for various extraction techniques (Datta et al. 2017). For industrial‐scale isolation of the main azadirachtins and additional bioactive meliacins, several liquid chromatographic modifications have been used, including column chromatography, reverse phase medium, thin layer chromatography, high‐pressure preparative liquid chromatography, and vacuum liquid chromatography (Hodgson et al. 2019).

Over time, various methods have been developed to prepare azadirachtin concentrates with different potencies for domestic and industrial applications. The indigenous techniques include water extraction, steam distillation, cold maceration, and organic solvent extraction. In a conical flask, defatted kernels are submerged in methanol during the maceration process, which is a cold‐extraction technique. After 3 days of intermittent stirring, the combination resulted in a dried crude extract, which was then filtered and, under vacuum, the solvent evaporated. A magnetic pellet was placed into the flask during batch stirring extraction, and the liquid was shaken on a magnetic stirrer plate for 8 h. The crude extract was prepared by vacuum evaporation of the solvent. Aqueous extracts may be produced with minimal equipment and are as effective as other methods. The cold extract can be utilized in the home after being prepared from powdered seeds, kernels, or leaves after being soaked and filtered overnight (Kumar et al. 2003).

An alternative method involves soaking the powder in a bucket overnight and steeping it using a cotton bag. The second option is to offer an extract. It usually includes several substances, carbohydrates, and amino acids dissolved in water. Compared to water, alcohol is a solvent for azadirachtin and other limonoids. Alcoholic extracts may contain approximately 0.26% of ingredients. They are generally 50 times more concentrated than the water extracts. The process of extraction with alcohol may resemble that of extraction with water (Table 4) (Chaudhary et al. 2017). Table 4 depicts the extraction techniques of neem and their utilization.

**TABLE 4:** Extraction techniques of neem and their utilization.

Method	Description	Utilization	References
Water Extraction	The process involves boiling neem leaves or seeds in water and separating the extract through filtration. This technique utilizes water as a solvent to extract water‐soluble components from neem.	Neem extracts are utilized as organic insecticides, effectively repelling insects and pests from crops Face masks, tonics, and cleansers use neem water extract due to its potent antibacterial and antifungal properties Neem water extracts are utilized in shampoos and hair treatments to deal with dandruff and enhance scalp well‐being	Hashim et al. (2021)
Solvent Extraction	This technique entails the dissolution of neem plant components in an appropriate solvent to extract lipophilic substances, including oils and other bioactive elements.	Manufacturers use neem oil from solvent extraction to make soaps, shampoos, lotions, and detergents Neem oil extracts produced using solvent extraction are used in pharmaceutical production to treat diverse conditions, including skin problems, infections, and inflammation Cosmetics use neem oil for its hydrating and antimicrobial properties	Susmitha et al. (2013)
Cold Pressing	Cold pressing is a method that uses mechanical pressure to extract oil from neem seeds while maintaining the integrity of its natural qualities.	Neem oil extracted by the cold‐pressing method is of superior quality and maintains a higher concentration of its inherent characteristics than alternative extraction techniques. It finds application in skincare products, hair oils, and medical formulations.	Hussein et al. (2021)
Steam Distillation	This technique employs steam to vaporize and transport essential oil molecules from neem plant components, which are subsequently condensed and gathered.	Neem essential oil is used in aromatherapy because it is tranquilizing and alleviating Because of its unique scent, manufacturers use neem essential oil to create perfumes and scents Mosquito coils, sprays, and creams use neem essential oil as an effective natural insect repellent	Babatunde et al. (2019)
CO2 Extraction	This technique utilizes supercritical CO_2_ as a solvent to extract lipophilic chemicals from neem plant materials, resulting in the production of extracts of superior quality.	Because of its therapeutic qualities and gentle formulation, skincare and haircare products use CO_2_‐extracted neem oil Pharmaceutical preparations use CO_2_‐extracted neem oil because of its high purity and effectiveness in treating various health conditions.	Hazarika et al. (2023)
Supercritical Fluid Extraction	This technique uses supercritical fluids, which possess characteristics of gases and liquids, to extract bioactive chemicals from neem plant components in a regulated manner.	Supercritical fluid extraction produces neem oil with superior purity, making it suitable for pharmaceutical and cosmetic applications Neem oil obtained through supercritical fluid extraction is used to produce herbal medicines for its therapeutic effects on the skin and overall health	Chaudhary et al. (2021)

## 6 Medicinal Uses

### 6.1 Ayurveda

Neem trees have been one of the major ingredients of traditional Indian Ayurvedic treatment since the prehistoric days. Neem bark, leaf, and oil extracts are used to treat various ailments, such as leprosy, constipation, and intestinal helminthiasis. Furthermore, it is vital to treat indolent ulcers, rheumatism, and chronic syphilitic sores (Kumar and Navaratnam 2013).

Neem oil is commonly used to treat various skin ailments. Blood morbidity, itching, skin ulcers, burning sensations, biliary disorders, and phthisis can be cured by combining fruits, bark, leaves, flowers, and roots. Young fruits and root barks are substitutes, antiperiodic, and tonic. Green twigs are used as toothbrushes and as preventive measures for teeth and mouth problems. Snake bites and scorpion stings are treated with bark, gum, leaves, and seeds. The bark treats nausea and vomiting and is astringent, bitter tonic, antiperiodic, and antipyretic (Sidat et al. 2023).

The gum is an effective demulcent for catarrhal conditions. Boils can be treated with a poultice made of leaves. Leaf decoction has been used as an antimicrobial agent for the treatment of dermatitis and ulcers. Dry flowers have stomach‐like effects. Seed oil has stimulating, antibacterial, and alterative properties against rheumatism and skin conditions. Many toothpastes and tooth powders contain neem bark as an active element because neem bark kills germs. Dentistry is often used to treat gum problems and to keep teeth healthy. Neem oil can effectively treat leprosy and other skin conditions (Raja and Devarajan 2023).

Neem is always combined with other herbs in the Ayurvedic medical system to increase its potency and improve its flavor. Licorice, sugar, honey, lemon juice, and spices such as cardamom are pitta‐balancing herbs that can be added to increase effectiveness or reduce adverse effects. Herbs and spices, such as orange peel, cinnamon, fennel seed, and licorice root, can also temper bitterness. In modern Ayurveda, neem has been recommended for diabetes mellitus, possibly by increasing the insulin receptor sensitivity (Kalaskar et al. 2021).

### 6.2 Homeopathy

In Homeopathy, the neem treats rheumatic discomfort, extremity pain, hand and foot pain, and sternal and rib pain. In addition, it is used to treat scabies, pemphigus, and eczema. It is also used for healing properties, such as reducing inflammation, fighting infections, and killing fungi and germs. People believe that Neem can assist with health issues such as diseases, fever, stomach problems, and skin conditions such as psoriasis, eczema, and acne (Gupta 2022). However, it is crucial to note that further research is required to comprehend the safety and efficacy of neem in remedies, despite its history in medicine and some encouraging scientific data suggesting its potential health benefits.

### 6.3 Unani

Neem is used as a blood purifier and re‐solvent. The leaves cure urinary tract sores and expel the wind. It is used to treat skin conditions and as an emmenagogue. Fruit serves as an astringent in bronchitis and leprosy (Reddy and Neelima 2022).

## 7 Therapeutic Potential

**FIGURE 3:**
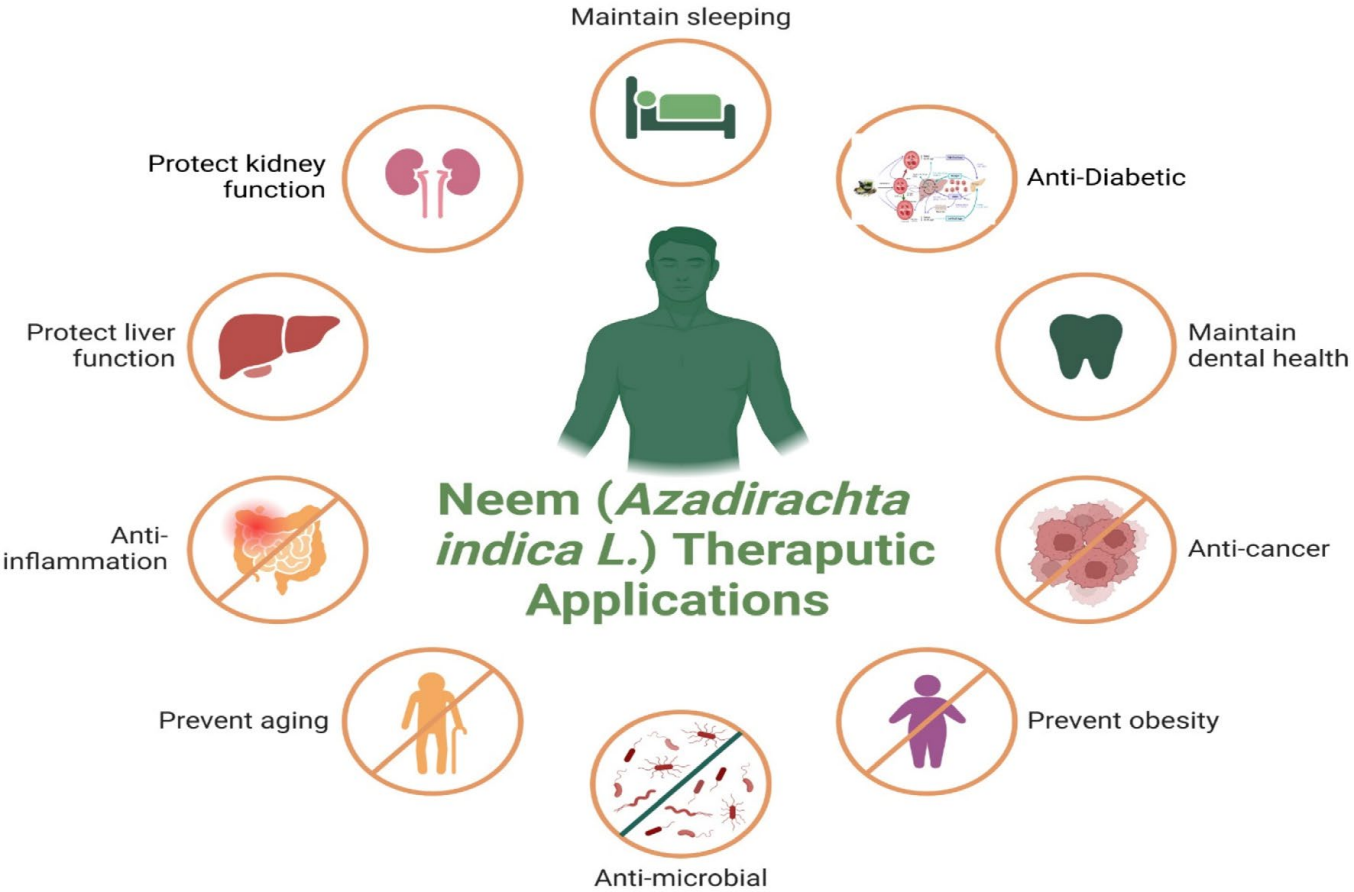
Therapeutic applications of neem.

Historically, numerous ailments have been treated using various portions of the neem tree. Today, many neem formulations sold in the market are used to treat illnesses (Figure 3). Numerous clinical and pharmacological studies have been conducted on various neem components and commercially available formulations. Almost universally, outcomes have been reported to be remarkably good.

### 7.1 Antioxidant

Reactive oxygen species (ROS) and free radicals are among the main factors leading to many diseases. One of the most important things you can do to stay healthy is to prevent free radicals from causing damage. Antioxidants prevent free radicals from damaging living cells by stabilizing them and rendering them inactive (Olajide et al. 2022). In addition, antioxidants help turn on an enzyme that prevents free radicals and ROS from causing damage (Liang et al. 2023). According to Bratovcic (2020), medicinal plants have been found to have antioxidant properties. Fruits, leaves, oil, bark, seeds, and roots are parts of plants that are high in antioxidants and lower the risk of getting sick. A study on the antioxidant activity of *A. indica* leaf and bark extracts revealed that all of the tested extracts, which are parts of neem plants that grow in the foothills, have high antioxidant activity (Itoh et al. 2020).

Other findings have indicated that nimbolide, azadirachtin, and ascorbate have concentration‐dependent free radical scavenging activity and reduction potential in the following order: nimbolide > azadirachtin > ascorbate. Azadirachtin and nimbolide treatment also prevented procarcinogen activation, oxidative DNA damage, and growth of antioxidant and carcinogen detoxification enzymes, which in turn prevented the formation of DMBA‐induced HBP carcinomas (Nagini et al. 2021). According to Awada et al. (2023), the investigation results demonstrated that root bark extract has a higher degree of free radical scavenging activity, with 50% scavenging activity at 27.3 μg/mL. The total antioxidant activity was 0.68 mM of ascorbic acid standard (Bangar et al. 2024).

### 7.2 Anticancer

Health is endangered by intricate cancer. Alterations in genetic and molecular mechanisms induce cancer. Allopathic therapy benefits one aspect while damaging healthy cells (Moga et al. 2018). Flavonoids and other constituents included in neem are essential for cancer prevention. Multiple epidemiological studies have indicated a correlation between heightened flavonoid consumption and a reduced risk of cancer Santos et al. (2023).

Neem oil contains a variety of limonoids that mitigate the mutagenic effects of 7,12‐dimethylbenz(a)anthracene (Lodi et al. 2025; Nagini et al. 2024). Human choriocarcinoma (BeWo) cells were used to examine the cytotoxic activity of nimbolide, which is present in leaves and flowers. The analysis indicates that nimbolide has proven successful in dose‐ and time‐dependent growth inhibition in BeWo cells, with IC50 values of 2.11 and 1.29 M for 7 and 24 h, respectively (Bhamare et al. 2020).

**FIGURE 4:**
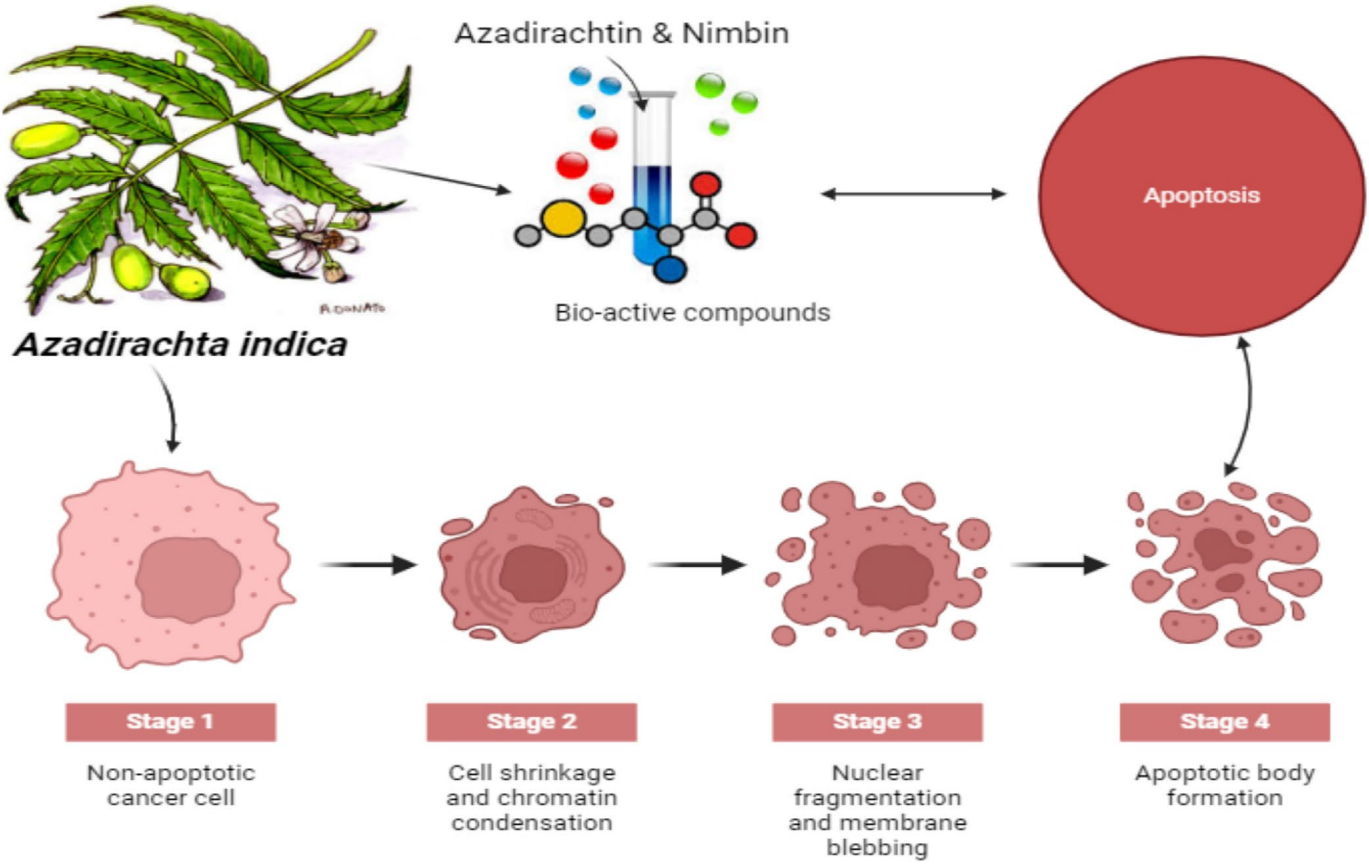
Neem's anticancer mechanism action.

A study found that nimbolide and azadirachtin prevented DMBA‐induced HBP carcinomas by preventing procarcinogen activation, oxidative DNA damage, antioxidant and carcinogen detoxification enzymes, tumor invasion, and angiogenesis (Nagini et al. 2021). *Azadirachta indica* and its bioactives dramatically reduce cancer. This method lacks molecular interaction certainty. Neem components may influence cell‐signaling pathways. Numerous *Azadirachta indica* components activate and deactivate tumor suppressor genes (Figure 4) (Agrawal et al. 2020).

Chemicals in *Azadirachta indica* inactivate cancer‐related genes including VEGF, NF‐B, and PI3K/Akt and activate tumor suppressor genes. Previously, neem was shown to promote tumor suppressor genes and inhibit VEGF and phosphoinositide PI3K/Akt pathways. It also activates cyclooxygenase, reduces NF‐B signaling, and promotes apoptosis (Dutt et al. 2023).

#### 7.2.1 Apoptosis

The apoptotic process requires Bcl2 and Bax. Changes in bcl2 and bax promote tumor development. Various cancers affect these genes' activity. In a mouse in vivo 4 T1 breast cancer model, the extract increased apoptosis rates in the CN 250 and CN 500 groups compared to cancer controls. A previous study shows that the extract induces apoptosis in prostate cancer cells PC‐3, killing them (Ibrahim et al. 2022).

Neem leaf extract dose‐dependently decreased chronic lymphocytic leukemia (CLL) cell viability, with significant cell mortality at 0.06% (w/v) during 24 h. Leaf extract also increased Bim, caspase‐8, and caspase‐3 expression, suggesting it induces apoptosis at the receptor. Neem's main components and separated chemicals influence several locations and activate cancer cell death via apoptosis (Nagini et al. 2021).

#### 7.2.2 Angiogenesis

Tumor development depends on angiogenesis, a complex process that supplies blood to tissue. Both activators and inhibitors control angiogenesis. Antiangiogenic medications that limit blood vessel expansion are essential to decreasing tumor growth. Medicinal herbs and their components prevent tumor growth by inhibiting angiogenesis (Ibrahim et al. 2022).

The ethanolic fraction of neem leaves as EFNL effectively lowers proangiogenic genes including vascular endothelial growth factor A and angiopoietin, according to extensive studies. Recent research suggests that the ethanolic fraction of neem leaf (EFNL), which inhibits angiogenesis, may reduce breast tumor volume and stop tumor progression (Azam et al. 2023).

Another study used HUVECs to examine the leaf extract's antiangiogenic effects. HUVECs treated with EENL showed decreased invasion, proliferation, and migration in vitro and VEGF‐induced angiogenic response in vivo and in lab testing. A study on growing zebra fish employed different quantities of methanolic crude extract of water‐soluble neem root fractions, imatinib (standard), and a control. Results revealed the extract may reduce angiogenesis (Hoseinkhani et al. 2020).

### 7.3 Anti‐Ulcer

According to a clinical trial, nimbidin, a bitter component of neem, is highly beneficial for treating duodenal ulcers and reducing discomfort in the epigastric area. Palmitic acid and stearic acid (fatty acids) are found in the nimbidin component of neem oil (in seeds). Therefore, these two fatty acids are thought to be crucial for the ulcer‐healing ability of nimbidin (Khanpara and Mavani 2022).

Highly effective antiacid secretory and anti‐ulcer action has been discovered in an aqueous neem bark extract (NBE), and the bioactive component has also been linked to a glycoside. Neem is one of the ingredients of the medication “Bhunimbadi Ghanasar,” which is particularly efficient in treating the signs of “amlapitta” (acid dyspeptic illness) rather than causing any adverse effects. The crude extract, “Nimbatiktam,” is obtained directly from neem oil or seed kernels. The active ingredient was nimbidin (1.2% w/w). The medication effectively treats ulcers without causing adverse effects (Kumar et al. 2022).

Salanin, a liminoid bitter component of neem seed oil, also possesses ulcer‐healing properties. Salamin showed protective efficacy against aspirin‐induced stomach lesions in test animals at oral doses of 50, 10, and 20 mg/kg. The effectiveness of NBE as a prospective healing agent for treating stomach ulcers and hyperacidity and its standardization, safety assessment, and mode of action were recently explored by Wakodkar et al. (2021).

### 7.4 Anti‐HIV/AIDS

Neem has a remarkable capacity to manage diseases that are directly transmitted. Neem provides 75% protection against human immunodeficiency virus (HIV) infection (Soni et al. 2024). A 12‐week oral course of (neem leaf) extract acetone water (IRAB) in HIV/AIDS patients significantly affected CD4 cells (which HIV decreases) in vivo without causing any adverse side effects. Fifty patients who received therapy and completed the study complied with all laboratory test requirements. In 50 patients, the average CD4 count increased by 159%. This is a notable improvement; the number of HIV/AIDS pathologies decreased from 120 baseline to 5, and significant gains were seen in body weight (12%), lymphocyte differential count (24%), and hemoglobin concentration (24%) IRAB is recommended as part of a therapy plan for HIV/AIDS. Neem may aid in screening for AIDS or act as a treatment when taken as part of a neem leaf tea or as individual neem leaves (Uzzaman 2020).

Autade et al. (2015) investigated the impact of neem plant extracts on bacterial and fungal pathogens associated with HIV‐related opportunistic infections. Opportunistic infections mainly affect individuals with weakened immune systems and are the primary cause of mortality in HIV‐infected patients. The antimicrobial activity of the acetone and chloroform extracts from neem leaf, fruit, and bark was examined using the agar well diffusion method against eight bacterial and two fungal diseases. The neem bark and leaf extracts obtained using acetone and chloroform exhibited the most potent inhibitory effect compared to the other treatments. A concentration of 30 mg extract on the disc was sufficient to inhibit most pathogens. *Staphylococcus aureus* and *Pseudomonas aeruginosa* exhibited greater susceptibility to plant extracts than other microorganisms. Neem leaf, bark, and fruit plant extracts showed a higher inhibition frequency against *Candida albicans* than *Cryptococcus neoformans* , which was exclusively inhibited by NBE. The adverse effects of synthetic antibiotics can be mitigated by substituting them with therapeutic bioactive molecules that can be consumed indefinitely. Studies conducted on human cells have demonstrated that fractionated acetone water neem leaf extract effectively prevents the invasion of HIV‐1 and leads to a notable increase in CD4+ cell counts in a limited number of HIV/AIDS patients (Awah et al. 2011; Mbah et al. 2007; Udeinya et al. 2004).

Another study by David et al. (2017) examined CEM T‐cell damage from neem leaf extracts, Azadirachtin, and Limonene. The extracts were concentrated at 1 and 10 ppm, whereas the compounds were at 1 and 10 μm. 100% cell viability was attained with 1 ppm aqueous extract and 1 and 10 μm azadirachtin concentrations, similar to the control group (*p* > 0.05). For HIV infection prevention, the study assessed the aqueous extract at 1 ppm and azadirachtin at both levels using cytotoxicity data. Over 50% inhibition was observed, considerably different from the control group (*p* < 0.001). This study revealed azadirachtin and aqueous neem leaf extract protected HIV.

### 7.5 Hepatoprotective

Hepatoprotection without adverse effects requires key medicinal plant components. In rats, azadirachtin was tested for CCl4‐induced hepatotoxicity prevention. Hepatocellular necrosis was dose‐dependently decreased by azadirachtin pretreatment, according to histological and ultrastructural data. Additionally, greater doses of azadirachtin pretreatment partly normalized the rat liver (Baligar et al. 2014).

Neem's active component, nimbolide, protected rats' livers from CCl4 poisoning in another study. These findings show that nimbolide protects the liver from CCl4‐induced liver damage as well as silymarin. Additionally, neem leaf extract protected rats against paracetamol‐induced liver necrosis (Bharali et al. 2023). A study found that *Azadirachta indica* (AI) leaf extract significantly reduced histological changes and prevented protein, bilirubin, alanine aminotransferase, alkaline phosphatase, and aspartate aminotransferase increases in antitubercular drug‐induced hepatotoxicity. It also stopped biomarker blood levels from rising (Gill et al. 2020).

Further experiments indicated that ethanolic and aqueous *A. indica* leaf extracts only moderately affected carbon tetrachloride‐treated mice. Methanolic and aqueous *Azadirachta indica* leaf extracts may protect the liver in rats. Tested for ethanol protection of rat stomach ulcers, neem extract was used. According to Sunday et al. (2024), the results showed that pretreatment with neem extract protects against the harmful effects of ethanol on the stomach mucosa.

### 7.6 Antimalarial

Neem contains antimalarial properties. Neem extracts have considerable impacts on Plasmodium falciparum, the human malarial parasite, growth and development in vivo and in vitro. The fact that neem components have antiplasmodium actions on parasites that traditional antimalarial drugs (pyrimethamine and chloroquine) do not affect suggests a novel mode of action (Mbugi et al. 2021).

Neem seed components are effective against both parasite stages that induce clinical manifestations and those that sustain malarial transmission. Limnoids (isomeldenin, meldenin, nimbandio, and nimocinol) derived from the ethanolic extract of fresh neem leaves are effective in combating the chloroquine‐resistant strain K1 of *P. falciparum* malaria (Akram et al. 2020).

Parasitemia in infected mice was reduced by 51%–80% and 56%–87% with leaf and stem bark extracts, respectively (Rahmah et al. 2024). Early erythrocytic schizogony in *P. berghei* infected inbred mice has also been examined for the effects of methanolic extracts of seed kernels from ripe and unripe neem fruits (Mwingira et al. 2023).

### 7.7 Antifungal

In vitro antifungal efficacy of seed kernels and neem leaves against *Penicillium expansum*, *Monilinia fructicola* , *Alternaria*, and *Trichothecium roseum*. Among aqueous, ethanolic, and ethyl acetate extracts, neem leaves have also shown noteworthy activity against *Aspergillus fumigatus* , *Microsporum gypseum*, *Aspergillus niger*, *Aspergillus flavus*, *Aspergillus terreus*, and *Candida albicans* . The highest antifungal effect was observed in the ethyl acetate and nimonol extracts, which were detected by HPLC analysis (Srivastava, David, et al. 2020; Srivastava, Agrawal, et al. 2020).

According to a recent study, using neem powder with acrylic resin denture base materials weakened *C. albicans* adherence and prevented denture stomatitis. An experiment was conducted to test the impact of several neem leaf extracts on the seed‐borne fungi, Rhizopus and Aspergillus. The results indicated that both aqueous and alcoholic extracts strongly suppressed and controlled the growth of both fungal species. Additionally, the neem leaf extract was more effective than the water extract in inhibiting the growth of these fungal species (Hamid et al. 2021).

Another study showed that three sporulating fungi, *C. lunata* , *H. pennisetti*, *C. gloeosporioides*, and *F. mangiferae*, were prevented from germinating by neem cake aqueous extracts antibacterial properties (Kumari et al. 2020). The results showed that an extract of *Azadirachta indica* in ethanol and methanol prevented the growth of *Cladosporium*, *Alternaria solani*, and *Aspergillus flavus*. The antifungal effects of *Azadirachta indica* on *Alternaria solani* were investigated. The outcomes demonstrated that the portion of ethyl acetate, with a MIC of 0.19 mg, was more efficient in reducing fungal growth and was also superior to the fungicide (mancozeb + metalaxyl), having a MIC of 0.78 micrograms (Adusei and Azupio 2022).

### 7.8 Antiviral

The neem plant ( *Azadirachta indica* ) possesses potent antiviral properties, rendering it a valuable natural remedy in mainstream and complementary healthcare (Petrera 2015). Neem leaves, bark, and seeds contain azadirachtin, nimbin, and quercetin, which have demonstrated potent antiviral effects against several viruses, including hepatitis B and C, herpes simplex virus, and HIV (Wylie and Merrell 2022). These bioactive compounds enhance the immune system, reduce the presence of viruses, and impede viral replication and penetration into host cells. The many antiviral characteristics of neem highlight its potential as a supplementary treatment for viral infections and to enhance overall immune function (Gadge 2021).

The findings demonstrated that NBE, at doses ranging from 50 to 100 μg/mL, effectively prevented HSV‐1 entry into cells (Garber et al. 2021). Neem bark directly inhibited HSV‐1 when cultured with the virus instead of target cells. Neem leaf extract (NCL‐11) reduces coxsackievirus B‐4 reproduction and is virucidal, according to virus inactivation and yield reduction tests (Joshi and Prabhakar 2021).

Tiwari et al. (2010) revealed that an aqueous neem plant Azadirachta indica bark extract prevented HSV‐1 from accessing its natural target cells. The NBE reduced HSV‐1 entry into cells at 50–100 μg/mL dosages. Pre‐incubating the extract with the virus showed NBE's inhibitory impact, but not with target cells. This shows that neem bark directly inhibits HSV‐1. NBE also prevented virions from binding to cells, suggesting it blocks attachment. NBE inhibits HSV‐1 glycoprotein‐induced cell fusion and polykaryocyte formation. It appears that NBE also prevents viral fusion.

### 7.9 Neurological Protection

Animal studies have also shown that standardized neem extract has neuroprotective properties. Allodynia, hyperalgesia, motor coordination, and motor nerve conduction velocity were significantly decreased in animal models (caused by partial sciatic nerve ligation) (Hu et al. 2023). However, prolonged use of this extract greatly lessens such behavioral abnormalities (Adetuyi et al. 2023). Additionally, when studied in animal models, neem extract dramatically decreased the heightened inflammatory mediators, oxidative and nitrosative stress impact, and expression of Bax and iNOS in mRNA. Patients frequently experience chronic neuropathic pain, a well‐known pain syndrome that is challenging for doctors to treat (Lozano et al. 2020).

*Azadirachta indica* exerts anti‐inflammatory, antioxidant, and analgesic effects. A study was conducted by Kandhare et al. (2017) to determine whether the standardized AI extract has any neuroprotective effects on peripheral neuropathy in an animal model induced by partial sciatic nerve ligation (PSNL). Male Wistar rats, weighing 180–200 g, were subjected to tight nerve ligation to induce PSNL. For 28 days, rats were administered either distilled water (PSNL control), AI (100, 200, and 400 mg/kg), or pyridoxine (100 mg/kg) for the evaluation of several behavioral, biochemical, molecular, and histological characteristics. While chronic therapy with AI (200 and 400 mg/kg) considerably reduced allodynia, hyperalgesia, motor coordination, and motor nerve conduction velocity (MNCV), PSNL dramatically increased these behavioral alterations. AI therapy also reduces the histological abnormalities caused by PSNL. *Azadirachta indica* improves MNCV by inhibiting oxidative‐nitrosative stress, producing pro‐inflammatory cytokines, and inducing apoptosis to exert its neuroprotective effects against PSNL‐induced neuropathic pain (Kandhare et al. 2017).

Cisplatin has been applied as a potential neurotoxic medication in mammalian model studies that elevate nitric oxide and lipid peroxidation levels, while lowering glutathione levels. NLE has also been examined for its neuroprotective benefits, along with other medicinal herbs, and has demonstrated extremely effective curative actions against cisplatin (Moneim 2014).

### 7.10 Antidiabetic

**FIGURE 5:**
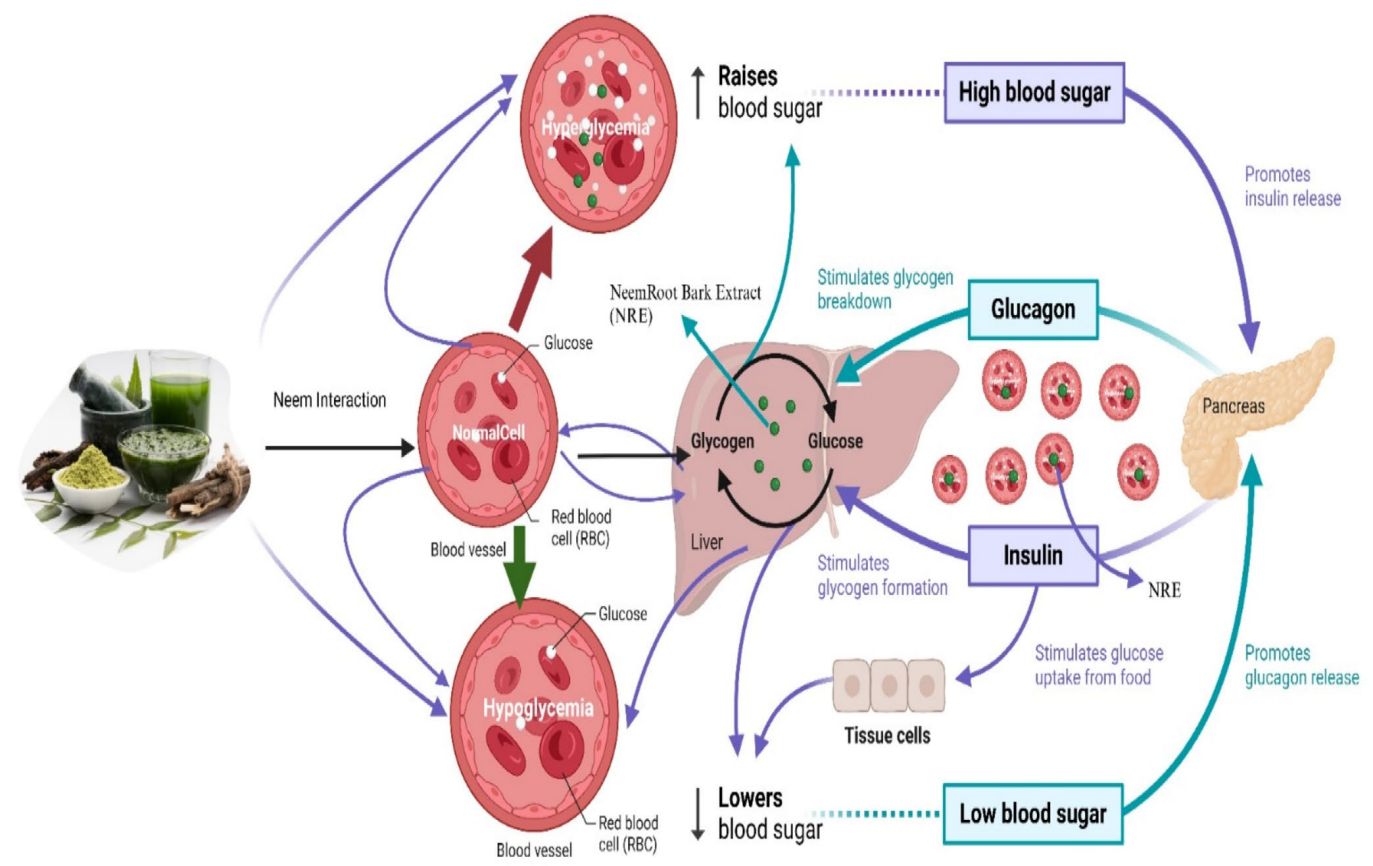
Antidiabetic mechanism action of neem.

In a trial to test neem root bark with 70% alcoholic extract, blood sugar levels were reduced at 200 and 400 mg/kg body weight. Blood sugar levels significantly decreased at higher doses of this extract (800 mg/kg body weight) and declined by 54% compared to the controls (Figure 5). Wistar albino rats of either sex that had been fasted overnight were assessed for basal blood sugar levels. In order to determine the antihyperglycemic effects of neem root bark extract (NRE), an oral glucose tolerance test (OGTT) was performed 60 min after the administration of the reference drug (glibenclamide), the test drug (NRE) at doses of 200, 400, and 800 mg/kg, and blood glucose levels were measured every half an hour for 4 h. Hypoglycemic activity was assessed in rats that had been administered equal doses of alloxan once a day for 15 days. Blood sugar levels were estimated (Patil et al. 2013).

Antidiabetic properties of neem tree extract were studied. In diabetic rats, *A. indica* was hypoglycemic. Neem extract at 250 mg/kg body weight reduced glucose levels more than the control group in a glucose tolerance test, whereas *Azadirachta indica* lowered glucose in hyperglycemic rats at 15 days (Dholi et al. 2011). Additionally, glibenclamide and neem kernel powder were employed as antidiabetic medications in experimental animals, either singly or in combination. The findings showed that these two medicines considerably lower blood glucose, fat, and enzyme concentrations when combined (Bopanna et al. 1997).

Studies utilizing methanolic, chloroform, and solvent extracts of *A. indica* and *B. spectabilis* were conducted using an in vivo diabetic mouse model. The results indicated that *B. spectabilis* aqueous and methanolic extracts, and chloroform *A. indica* extract demonstrated useful oral glucose tolerance and dramatically decreased the intestinal activity of glucosidase (Bhat et al. 2012).

Another noteworthy study revealed that *Andrographis paniculata* and *A. indica* leaf extracts might help treat type 2 diabetes owing to their strong antidiabetic properties. In both glucose‐loaded and alloxan‐induced diabetic rats, the hypoglycemic efficacy of the concentrated 90% ethanolic extract of *Andrographis paniculata* and *Azadirachta indica* was examined in comparison with that of a reference antidiabetic medication, glimepiride. The acute toxicity of the two plant extracts was investigated. According to experimental findings, *Andrographis paniculata* and *Azadirachta indica* ethanol leaf extract (1 g/kg) effectively lowered blood glucose levels by 40.65% and 36.91%, respectively, in rats administered glucose and by 32.18% and 30.20%, respectively, in rats administered alloxan to induce diabetes, when compared to the corresponding diabetic control group. This study indicates that *Andrographis paniculata* and *Azadirachta indica* ethanol leaf extracts have high antidiabetic activity and may be a source of diabetes mellitus recovery (Akter et al. 2021).

### 7.11 Wound‐Healing

**FIGURE 6:**
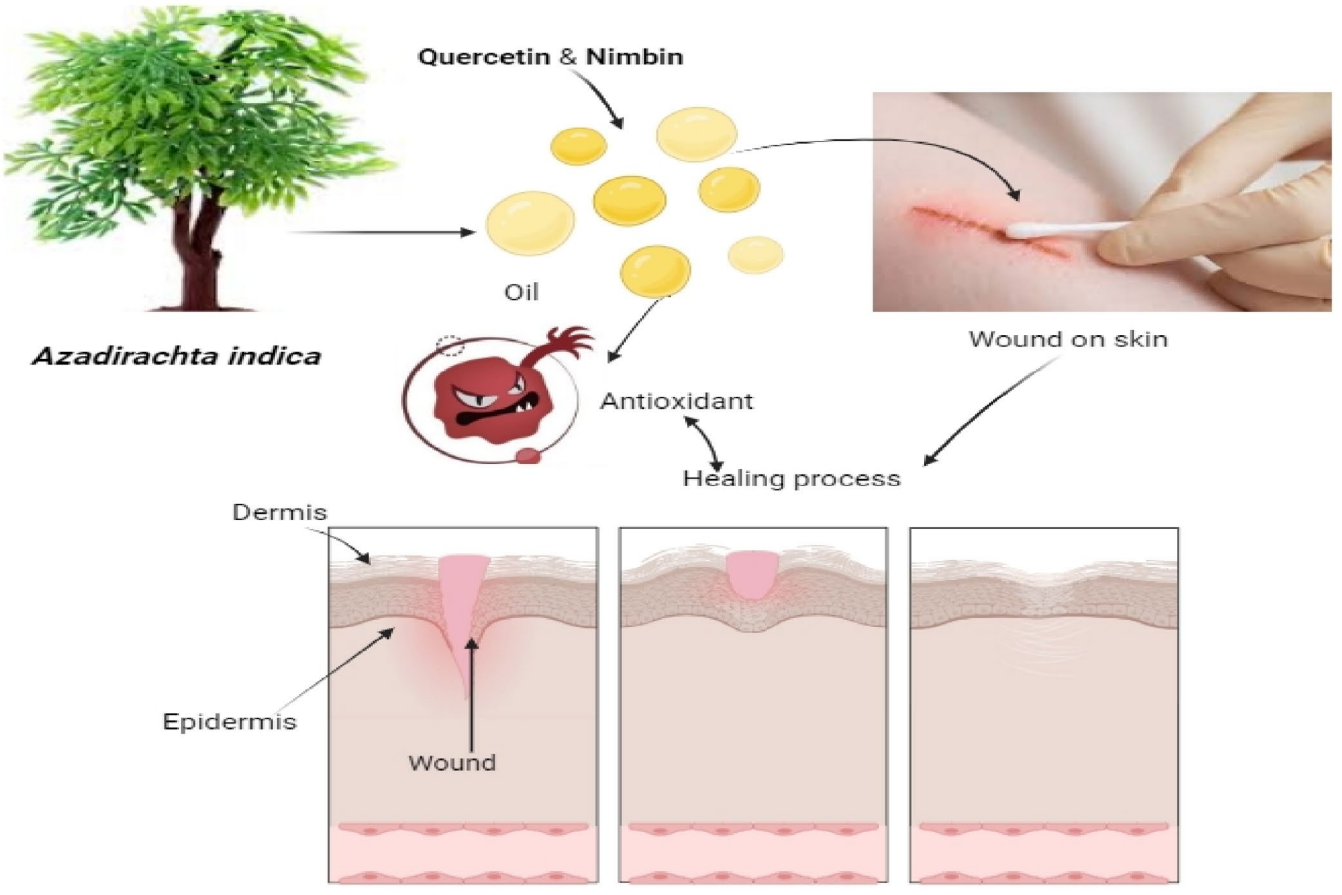
Wound healing mechanism action of neem.

Neem leaves have been used for centuries as a folk remedy for wound mending. The benefits of neem oil in treating chronic, unhealed wounds were examined in one study, and the findings revealed that after 8 weeks of therapy, over 44% of patients had 50% wound healing (Figure 6) (Singh et al. 2014).

An experimental investigation performed by Chundran et al. (2015) aimed to gauge the neem leaves capacity for healing. Neem leaves ( *Azadirachta indica* ) contain therapeutic compounds, such as sodium nimbidate and nimbidin, as well as good nutrition, which is essential for the production of collagen and the growth of new capillaries. Twenty‐seven rats were randomly divided into three groups, and an excision incision (1.5 cm) was made. The neem leaf extract was applied topically to the treatment group; the positive control group received a topical application of povidone‐iodine, and the negative control group received a saline solution topical application of sodium chloride 0.9%. The largest diameter of the raw wound surface acted as a sign of healing on days 0, 5, 10, and 15. Compared to the group that received the 0.9% NaCl treatment, there was a substantial decrease in the longest width of the wound in the neem leaf extract group, and there was no discernible difference between povidone‐iodine and neem leaf extract in the longest diameter of the wound (Zeng et al. 2020). According to research, the ability of neem leaf extract to heal wounds biochemically through neovascularization and an inflammatory reaction makes it effective (Alzohairy 2016).

### 7.12 Dentistry's Function

**FIGURE 7:**
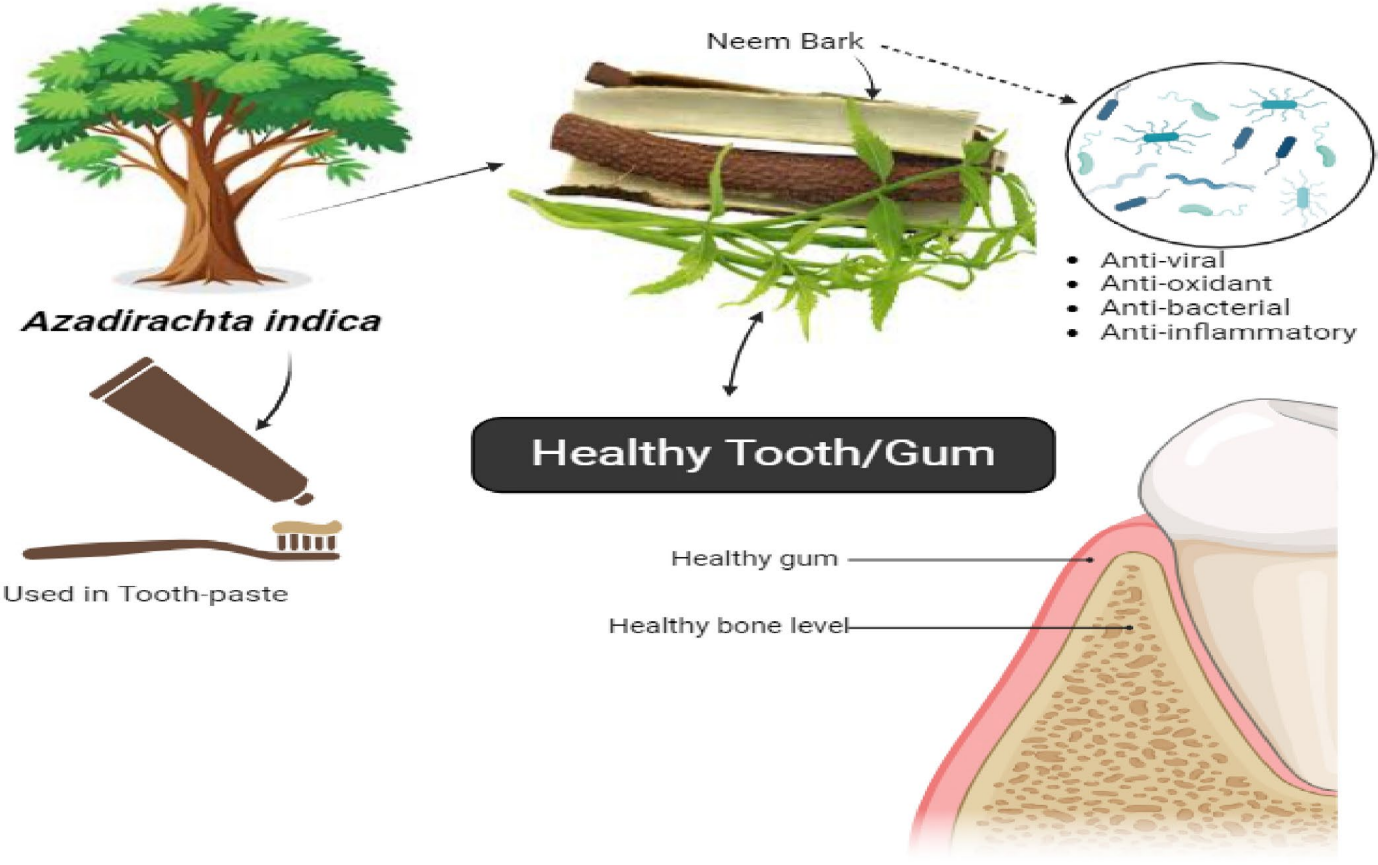
Neem role in teeth health.

The neem extracts produced using several organic solvents exhibited antibacterial activity (Figure 7). Neem sticks, commercial toothbrushes, and toothpaste were compared in a comparative study to examine how well they removed plaque and maintained gingival health. The findings showed that there was no discernible difference between the two tooth cleaning methods and that both significantly reduced plaque and gingival scores compared to the comparison group (Bhambal et al. 2011).

According to preliminary studies, the amount of plaque on teeth can be reduced by the daily application of neem leaf concentrate‐containing gel to the gums and teeth for approximately one and a half months. Additionally, it could reduce the number of germ cells that lead to plaque formation in the mouth. It is unclear whether using a mouthwash containing neem reduces plaque formation. The effectiveness of the antibacterial characteristics of neem extract against bacterial strains was assessed. The antibacterial efficacy of chloroform extract and petroleum ether against *Streptococcus mutans* has been proven by previous studies. The results also showed that the chloroform extracts have potent antibacterial properties (Dandekar and Winnier 2020).

Organic extracts of neem were prepared using a variety of solvents, including petroleum ether, chloroform, ethanol, and distilled water, and were then tested for their ability to kill microorganisms (Joel David 2021). Petroleum ether and chloroform extracts from neem outperformed the other three, with an inhibitory zone of 18 mm at 500 g against *S. mutans* . The neem extract in chloroform exhibited significant antimicrobial activity with an 18 mm inhibitory zone against *Streptococcus salivarius* . The inhibition zone of the 3rd strain of *Fusobacterium nucleatum* , which was hyperdelicate to both ethanol and neem water extract, was 16 mm. These findings show that neem chloroform extracts have potent antibacterial properties and may help manage dental caries (Lakshmi et al. 2015).

### 7.13 Antidermatophytic

Neem has a surprising effect on chronic skin diseases, which sometimes do not improve with medication. It has been discovered that lotion application locally made from 70% neem leaf alcoholic extract is beneficial in treating chronic skin conditions such as scabies, eczema, and ringworm infection (Giuggioli et al. 2020).

Salicylic and benzoic acids are less efficient in treating ringworms than alcoholic neem leaf extract. It has been shown that a 4:1 blend of fresh neem leaves and turmeric powder is useful for treating scabies and using neem leaf extract, which has antidermatophytic action against a variety of dermatophytes, namely *Trichophyton ruberum*, *Trichophyton violaceum*, *Trichophyton*, *Epidermophyton floccosum*, *Microsporum nanum*, and *Mentagrophytes* (Tiple et al. 2024).

## 8 Neem Toxicity Levels and LD50 Values

It is essential to test the toxicity of natural compounds prior to their use in health treatment. Neem is safe at specific doses according to research based on animal models and clinical trials, although neem and its constituents have hazardous or detrimental effects (Islas et al. 2020).

According to several investigations, neem oil intoxication causes vomiting, liver toxicity, metabolic acidosis, and encephalopathy in children. According to a rat model‐based investigation, administering leaf sap had an anxiolytic effect at low dosages but no such effect at higher levels. Significant research using rats as a model demonstrated that azadirachtin did not exhibit toxicity, even at 5 g/kg body weight (Raizada et al. 2001). Rabbit research was conducted to evaluate toxicological analyses. The study findings revealed that the body weights of test and control animals gradually increased. Neither group exhibited toxicity when neem extract was administered (Boadu et al. 2011).

According to a study's findings, neem oil LD50 values for acute toxicity tests were determined to be 31.95 g/kg (Deng et al. 2013). Another investigation into toxicity in chickens demonstrated that the clinical symptoms depend on the amount of the drug, and the intraperitoneal LD50 of neem leaf aqueous extract for acute poisoning was 4800 mg/kg (Biu et al. 2010). Neem leaf and stem bark extracts have fatal median doses (LD50) of 31.62 and 489.90 mg/kg body weight, respectively (Akin‐Osanaiya et al. 2013). Water extracts of the leaves and seeds of *A. indica* had LD50s of 6.2 and 9.4 mL kg^−1^, respectively (Bakr 2012). By using probit analysis, the LD50 and LD90 values of neem extract were determined to be 8.4 and 169.8 μg/fly, respectively. Acute oral toxicity tests in mice have shown an LD50 value of approximately 13 g/kg body weight (Khan and Ahmed 2000).

## 9 Neem's Multifaced Applications

### 9.1 Agricultural

Most scientists have concentrated their studies on agriculture to examine the advantages of neem in crop development. Neem oil, cake, leaves, and other neem tree components are now widely employed in the agricultural industry worldwide because of their discovery. Its primary agricultural applications are foliar pesticides, soil amendments, food storage insecticides, fertilizer efficiency enhancers, and soil amendments (Lokanadhan et al. 2012).

Pests can be managed without resorting to violence by using neem derivatives. Neem products interfere with the insect life cycle at various stages. They may not immediately kill the pest, but they may hinder it in several ways. Neem exerts effects such as stifling food and development, interference with mating, and chemical sterilization (Adusei and Azupio 2022).

### 9.2 Medicines

Fever is commonly treated with neem. It possesses antipyretic (fever‐reducing) properties. Neem products also have analgesic (pain‐reducing) and anti‐inflammatory actions, making them effective for most common illnesses. Neem provides affordable, conveniently accessible, and locally produced medications. Different neem components can be used to treat various illnesses or disorders (Giri et al. 2019). Herbal remedies are used to treat various disorders in multiple regions worldwide. The neem tree, which plays a significant role in various herbal remedies, has been referenced in ancient medicine literature (Bhamare et al. 2020).

Traditional Indian medical experts regard this as the best available remedy. Because of Neem's unique qualities, its bark, flowers, roots, leaves, seeds, and fruit pulp have been used to treat various illnesses and complaints, including leprosy, diabetes, ulcers, skin conditions, and constipation. Multiple companies, including the Himalayas, produce neem medications. To create new antibiotics, scientists from the industrialized world are actively researching neem trees and their qualities (Wylie and Merrell 2022).

### 9.3 Cosmetics

Numerous products, including skin creams/lotions, soaps, toothpaste, shampoos, beauty products, and toiletries are made from various neem tree components. Branches from the neem are the most frequently used antibacterial toothbrushes. Most of the time, neem oil or extract is used to create cosmetics such as soaps and toothpaste (Uzwatania and Ningrum 2020). High‐grade herbal cosmetics and beauty products frequently contain neem and its byproducts. Natural cosmetics are in high demand worldwide because of their effectiveness and lack of adverse effects. Body lotions, fairness creams, and hand creams made by reputable herbal product makers are in high demand in the US, the UK, and other countries. Neem oil and decoctions can be administered to the body with creams and lotions (Ahuja et al. 2021).

Additionally, it is used as a face cleanser, beauty booster, and radiant complexion. Numerous high‐quality herbal, cosmetic, and personal hygiene items have been produced and exported using neem bark. Creams and lotions for the face and body were made from powdered leaves. It is necessary to maintain skin glowing and healthy. The shampoo contains neem leaf extracts that reduce dandruff. Owing to their antibacterial and antifungal properties, leaf granules are utilized in herbal face packs (Bhamare et al. 2020).

### 9.4 Food Storage

Infestations by worms, beetles, and other pests cause a significant portion of the food produced in all tropical regions to spoil during storage. People do not want to use chemical pesticides or synthetic bug sprays on grains that are being kept, particularly food that is being preserved for use (Campos et al. 2016). Neem oil has long provided farmers with a powerful defense against these insects. With no degradation or loss of flavor, a very thin coating of neem oil can shield stored food crops from all pests for up to 20 months. Food grains are protected from pests by storage in the neem leaves. When stored, neem leaves are pesticides. Because they absorb moisture from grains, dried neem leaves are used to store food grains. Although this is done on a limited scale, neem fresh leaves are also used sparingly to properly preserve food grains at home (Elvira and Wuryandari 2023).

### 9.5 Soil Amendment

Neem cake refers to a substance that is still present after oil removal from seed kernels. For millennia, the Indian subcontinent has used neem cake as a powerful soil improver. Farmers in this area have discovered that adding neem cake to soil results in healthier, more valuable plants with little to no insect or disease problems (Rani 2022).

Several studies have also been conducted to determine why plants thrive in soil combined with neem cake. Their findings demonstrated that neem cake is more decadent in plant‐available nutrients than manure; it also eliminates harmful nematodes, encourages a large population of earthworms, helps maintain nitrogen availability for plants, and offers notable insect protection (Srivastava, David, et al. 2020; Srivastava, Agrawal, et al. 2020).

Nematodes suck fluid from plant roots to the point where the roots are unable to provide the plant with sufficient nutrition. Subsequently, despite having enough food, water, and care, the plants appear ill, fail to thrive, and may finally perish. Neem cake first shields plants from insects and pests, enabling them to build a powerful defense against pest attacks. Second, Neem components absorbed via soil strengthen these built‐in defensive mechanisms because of their well‐known nutritional, antifungal, and insect‐repelling qualities (Habib 2024).

### 9.6 Veterinarian Uses

Neem has been used to protect live animals for various reasons. Additionally, it has been extensively utilized as animal feed. The two brothers, Sahadeva and Nakul, who prepared remedies using neem oil and leaves to cure injured horses and elephants, are mentioned in the epic of the Mahabharata (3000 B.C.) (Shinde and Somani 2023).

Neem extracts with anti‐ulcer, antibacterial, and antiviral characteristics successfully treat intestinal helminthiasis, stomach worms, and ulcers. Animals can be treated with any component of the neem plant, including gum, bark, leaves, fruits, and seeds (Modi and Soni 2023). Neem leaves are mainly used as antiviral medications against viruses that cause Newcastle disease, vaccinia, variola, foulfox, and other viruses. Swollen glands, bruising, and sprains can all be treated with a hot infusion of leaves. Bark is useful for the treatment of skin issues. Neem oil lowers blood sugar levels (Wasim et al. 2023).

Setaria cervi, a cow filarial parasite, is killed by aqueous and alcoholic neem flower preparation. Neem has long been used to control insects that affect cattle, such as hornflies, maggots, biting flies, and blow flies. The animals were fed neem oil, de‐oiled neem seed cake, and neem leaves. Except for Zn, neem leaves contain many proteins, minerals, carotenoids, and trace minerals (Gopan et al. 2021).

Moreover, they comprise appreciable amounts of digestible crude protein (DCP) and total digestible minerals (TDMs). Neem leaves are fed to animals such as cattle, buffalo, goats, sheep, and camels. Neem oil, which is rich in long‐chain fatty acids, is incorporated in poultry feed. De‐oiled neem seed cake includes fiber, sulfur, nitrogen, and essential amino acids. The processed cake offers premium chicken feed and possesses a good aperitif and vermicidal effect (Oluwafemi and Oluwayinka 2020).

Neem leaves are also used in the poultry industry to combat aflatoxicosis, which is induced by *Aspergillus flavus* and caused by contaminated chicken feed. Neem leaf extract prevents the production of aflatoxin by *Aspergillus parasiticus* and patulin by *Penicillium expansum* (Khodaverdi et al. 2021).

## 10 Conclusion

*Azadirachta indica* and neem are members of the family Meliaceae. It accomplishes this by increasing antioxidant activity, inhibiting bacterial development, and appropriately modulating various biological processes. Neem and its bioactive constituents have been used therapeutically for centuries, particularly in India. The leaves, seeds, blossoms, and bark of this tree are widely used for various applications. Numerous phytochemicals have been isolated from different parts of the plant, including triterpenes, gallic acid, nimbins, saponins, catechins, limonoids, flavonoids, phenols, and glycoproteins. The leaves also contain a variety of active ingredients, but the most important active constituent is azadirachtin, while the others are sodium nimbinate, gedunin, salannin, quercetin, nimbin, nimbidin, and nimbidol. Clinical investigations have shown that neem is beneficial for the prevention and treatment of numerous diseases. The immunomodulatory, antiviral, anti‐ulcer, antioxidant, anti‐inflammatory, antihyperglycemic, antifungal, and anti‐carcinogenic properties of neem and its components are well known. It strengthens the immune system and helps to treat inflammatory skin conditions. Detailed clinical trials should be conducted in animals to determine the specific mechanisms of action in illness management.

## Author Contributions

**Tabussam Tufail:** methodology (equal), writing – original draft (equal). **Huma Bader Ul Ain:** formal analysis (equal), visualization (equal). **Aiman Ijaz:** conceptualization (equal), supervision (equal). **Muhammad Adnan Nasir:** investigation (equal), resources (equal). **Ali Ikram:** supervision (equal), validation (equal). **Sana Noreen:** conceptualization (equal), visualization (equal). **Muhammad Tayyab Arshad:** data curation (equal), writing – review and editing (equal). **Muhammed Adem Abdullahi:** project administration (equal), writing – original draft (equal).

## Disclosure

The authors have nothing to report.

## Consent

This study did not involve humans.

## Conflicts of Interest

The authors declare no conflicts of interest.

## Data Availability

The data supporting the findings of this study are available from the corresponding author upon reasonable request.
